# Keep Eyes on Integrins

**Published:** 2016-02-27

**Authors:** Zhichao Fan, Wei Liu

**Affiliations:** 1 Division of Inflammation Biology, La Jolla Institute for Allergy & Immunology, La Jolla, CA 92037, USA; 2 Cell Death and Survival Networks Program, Sanford Burnham Prebys Medical Discovery Institute, La Jolla, California, USA

## Integrins

Integrins are conformation-regulated adhesion molecules, which play essential roles in many biological processes, especially for immunity [1]. Structurally, integrins are a group of heterodimeric transmembrane receptors, which contain α subunit and β subunit.

**Figure 1:**
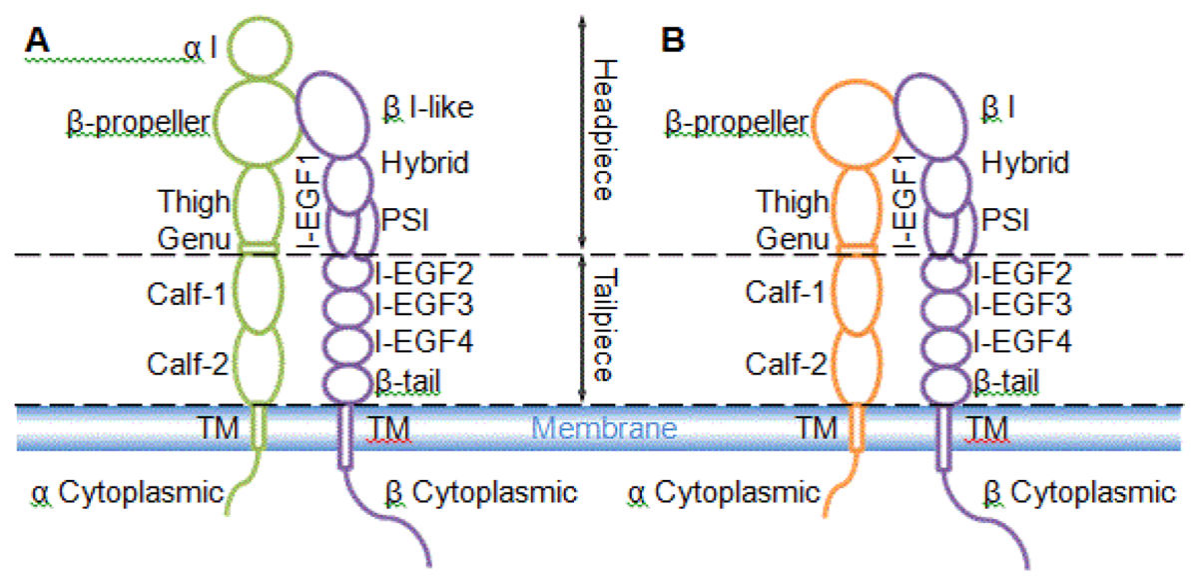
Structural schematic of the extended integrins. (A) Integrins with α I domain. α chain green, β chain purple; (B) Integrins without α I domain. α chain orange, β chain purple; Subdomains and headpiece/tailpiece portions labeled. TM means transmembrane domain.

In mammals, there are 18 kinds of α subunit and 8 kinds of β subunit were discovered, which form 24 combinations (Table 1) [2,3]. Overall, these 24 kinds of integrins can be divided to two groups by containing α-I domain (Table 1 and Figure 1A) or not (Table 1 and Figure 1B). Integrin can regulate its ligand-binding affinity by conformational changes [1]. The adaptor proteins and signal pathways were broadly studied, as discussed in ref 1 [1].

**Figure 2:**
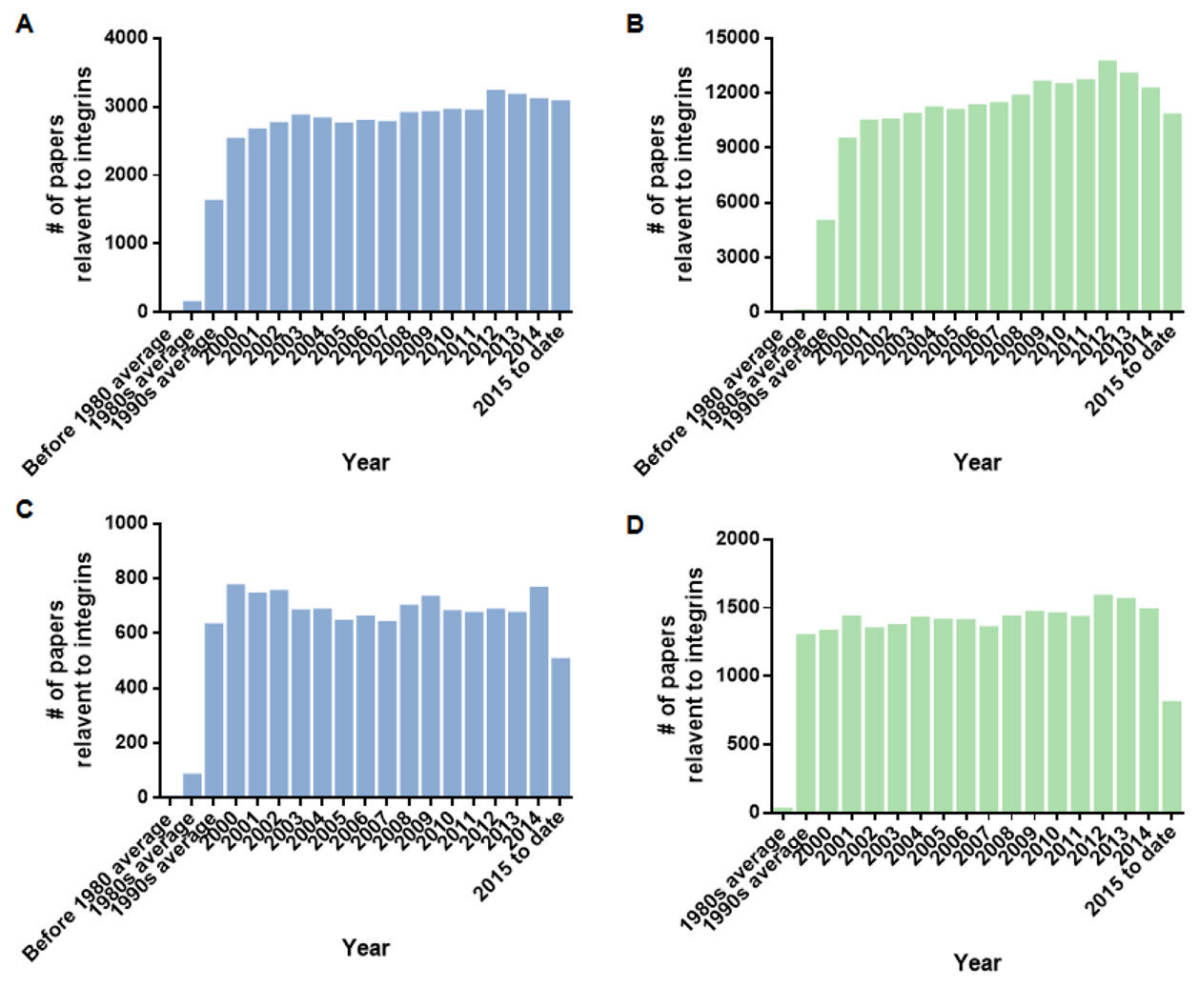
Statistics of the publications relevant to integrins. (A) Data showing the number of integrin-relevant publications per year pulling out from the PubMed; (B) Data showing the number of integrin-relevant publications per year pulling out from the Web of Science; (C) Data showing the number of integrin-relevant publications per year in the immunology catalog pulling out from the PubMed; (D) Data showing the number of integrin-relevant publications per year in the immunology catalog pulling out from the Web of Science.

Integrins are one of the most important adhesion molecules mediating cell-cell interactions and cell-extracellular matrix interactions. In immunity, integrins play critical roles in leukocyte adhesion from blood flow [1,4–6] cell migration [7], immunological synapse [8,9] and phagocytosis [10]. Thousands of scientific studies (PubMed: 63538 total, 18067 in immunology catalog; Web of Science: 236304 total, 52308 in immunology catalog) were presented up to data. When plotting the number of integrin studies by time (Figure 2), we can see the number of integrin-relevant studies was raised at 1980s, and become more popular in 1990s. In the last 15 years, integrin studies were continuously popular and got the most publications came out at 2012 (Figures 2A and B). When we see this statistics in immunology catalog, they presented similar trends (Figures 2C and D). Unfortunately, there’s a remarkable number decrease of the integrin-relevant paper in the field of immunology from 2015 to date. This indicates that integrin study reach a bottleneck after the boom when techniques of structure biology [11,12] and advanced microscopy [13,14] were introduced into the integrin field.

**Figure 3:**
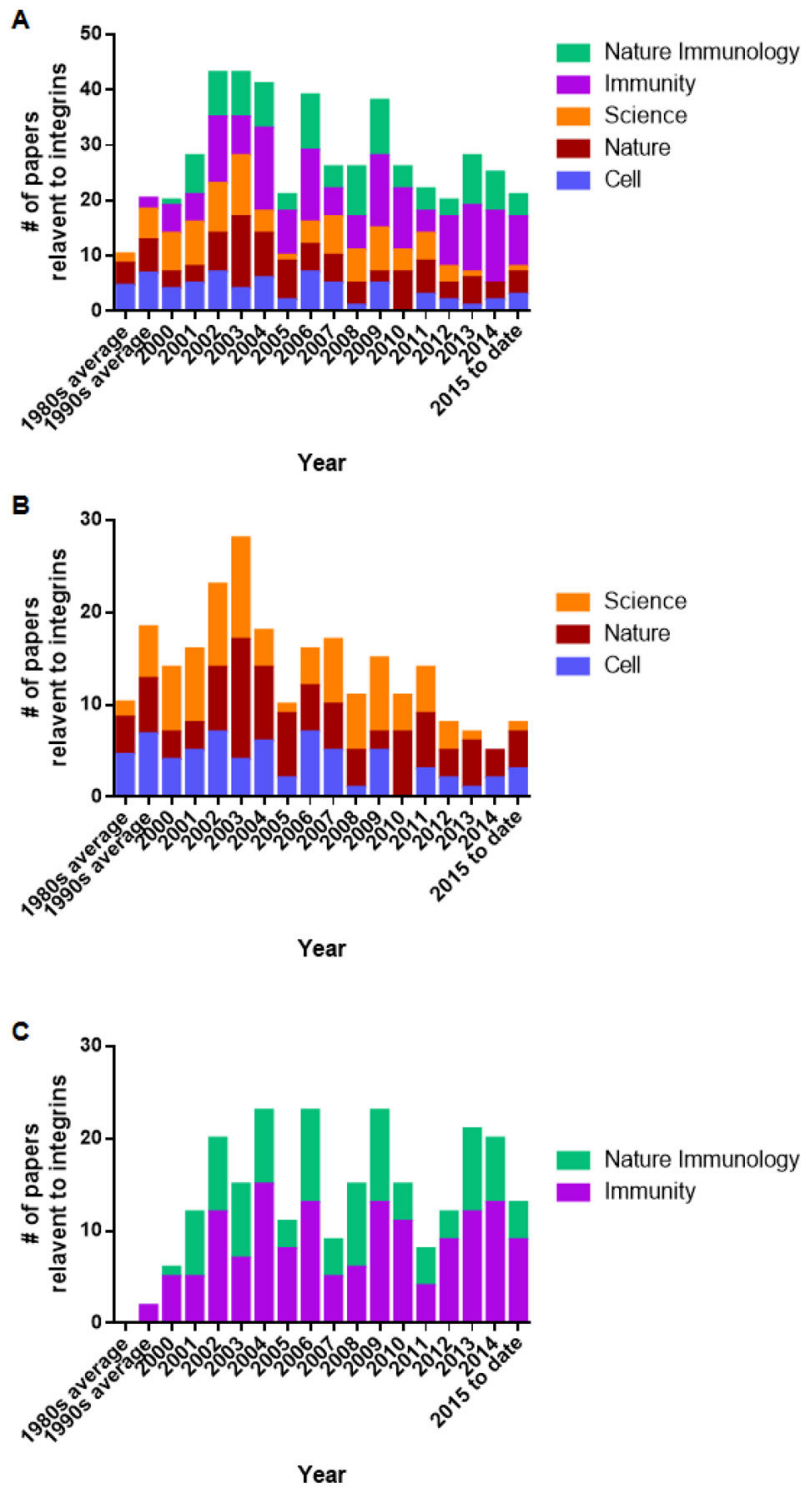
Statistics of publications relevant to integrins on top immunology-relevant journals. (A) Data showing the number of integrin-relevant publications per year in Nature, Science, Cell, Immunity and Nature Immunology; (B) Data showing the number of integrin-relevant publications per year in Nature, Science, and Cell; (C) Data showing the number of integrin-relevant publications per year in Immunity and Nature Immunology.

When focusing on the number of integrin studies on top-ranked journals in the field of immunology (5 journals were selected here including Nature, Science, Cell, Immunity and Nature Immunology), it presented that integrin study became a cutting-edge at early 2000s, most hot in 2002–2004, and continuously being concerned after that (Figure 3A).

When separating the integrin publication number from Nature, Science and Cell (representing the broad influences to the frontier scientific field, figure 3B) with that from Immunity and Nature Immunology (representing the influences to the frontier immunology field, figure 3C), it showed that integrins were quite popular in the frontier scientific field around 2003 (Figure 3B), and relatively lost concerns after 2010. One the other hand, integrin studies were popular from 2002 to 2014 in the frontier immunology field, whereas the manias seemed taking over after 2015.

In conclusion, integrin studies are keep concerned from late 1980s, and came to the forefront of the scientific community in early 2000s by introducing frontier techniques. Over the past decade, many studies about the regulation of integrin structure, pathway of integrin activation and integrin roles in immunity were emerged and made integrin as the most understood adhesion receptors. However, it seems that integrin enthusiasm was faded away since 2015. Actually, although integrins were vast investigated, there are many controversies, questions and holes remain. Immunologists, it is far away to see goodbye to integrins and please keep your eyes on them.

## Figures and Tables

**Table 1:** Type of integrins discovered,

α subunit (CD name)	β subunit (CD name)	Alternative name
α_1_ (CD49a)*	β_1_ (CD29)	VLA-1
α_2_ (CD49b)*	β_1_ (CD29)	VLA-2
α_3_ (CD49c)	β_1_ (CD29)	VLA-3
α_4_ (CD49d)	β_1_ (CD29)	VLA-4
α_5_ (CD49e)	β_1_ (CD29)	VLA-5
α_6_	β_1_ (CD29)	VLA-6
α_7_	β_1_ (CD29)	N.A.
α_8_	β_1_ (CD29)	N.A.
α_9_	β_1_ (CD29)	RLC
α_10_*	β_1_ (CD29)	N.A.
α_11_*	β_1_ (CD29)	N.A.
α_L_ (CD11a)*	β_2_ (CD18)	LFA-1
α_M_ (CD11b)*	β_2_ (CD18)	Mac-1
α_X_ (CD11c)*	β_2_ (CD18)	p150, 95
α_D_ (CD11d)*	β_2_ (CD18)	N.A.
α_IIb_ (CD41)	β_3_ (CD61)	GPIIbIIIa
α_V_ (CD51)	β_3_ (CD61)	vitronectin receptor
α_6_	β_4_ (CD104)	N.A.
α_V_ (CD51)	β_5_	N.A.
α_V_ (CD51)	β_6_	N.A.
α_4_ (CD49d)	β_7_	LPAM-1
α_E_ (CD103)*	β_7_	HML-1
α_V_ (CD51)	β_8_	N.A.

* These α subunits have α I domain.

CD means cluster of differentiation; VLA means very late activation antigen; RLC means regulatory light chains; LFA means lymphocyte function-associated antigen; Mac means Macrophage antigen; GP means glycoprotein; LPAM means lymphocyte Peyer’s patch adhesion molecule; HML means Human intestinal lymphocyte; N.A. means not applicable.
